# Antibacterial Structure Design of Porous Ti6Al4V by 3D Printing and Anodic Oxidation

**DOI:** 10.3390/ma16155206

**Published:** 2023-07-25

**Authors:** Guijun Yang, Houjiang Liu, Ang Li, Tiansheng Liu, Qiqin Lu, Fang He

**Affiliations:** 1 School of Materials Science and Engineering, Tianjin University, Tianjin 300072, China; junyygg@126.com (G.Y.); hj_liu@tju.edu.cn (H.L.); aliar@connect.ust.hk (A.L.); 2 College of Chemical Engineering, Qinghai University, Xining 810016, China; 3 Tianjin Hospital, Tianjin University, Tianjin 300211, China

**Keywords:** Ti6Al4V porous titanium alloy, 3D printing, anodic oxidation, antibacterial

## Abstract

Titanium alloy Ti6Al4V is a commonly used bone implant material, primarily prepared as a porous material to better match the elastic modulus of human bone. However, titanium alloy is biologically inert and does not have antibacterial properties. At the same time, the porous structure with a large specific surface area also increases the risk of infection, leading to surgical failure. In this paper, we prepared three porous samples with different porosities of 60%, 75%, and 85%, respectively (for short, 3D-60, 3D-75, and 3D-85) using 3D printing technology and clarified the mechanical properties. Through tensile experiments, when the porosity was 60%, the compressive modulus was within the elastic modulus of human bone. Anodic oxidation technology carried out the surface modification of a 3D-printed porous titanium alloy with 60% porosity. Through change, the different voltages and times on the TiO_2_ oxide layer on the 3D-printed porous titanium alloy are different, and it reveals the growth mechanism of the TiO_2_ oxide layer on a 3D-printed unique titanium alloy. The surface hydrophilic and antibacterial properties of 3D-printed porous titanium alloy were significantly improved after modification by anodic oxidation.

## 1. Introduction

In the biomedical field, titanium alloy has been utilized as an implant material since the 20th century due to its remarkable mechanical properties, favorable biocompatibility, and excellent corrosion resistance, resulting in a higher clinical bone repair effect. For decades, it has been successfully utilized for manufacturing artificial dental and orthopedic implants [1,2,3,4]. Nonetheless, the elastic modulus of titanium alloy is significantly larger than that of the human bone, which makes it easy to cause a “stress shielding” effect after implantation, leading to osteoporosis and a loss of bone around the implant, which increases the risk of bone implant failure [5]. Compared to Ti-based implants, porous titanium scaffolds have suitable mechanical properties and promote bone growth [6]. Many domestic and foreign scholars have studied the different porous structures. They have demonstrated that such systems may help the division and proliferation of osteocytes through animal experiments in vivo and cell experiments in vitro [7,8,9,10]. Due to significant developments in 3D printing technology, much more intricate and varied porous structures have become available [11]. However, there is currently limited research regarding the surface modification of 3D-printed porous titanium alloy, especially its antibacterial properties. 

Periprosthetic Joint Infection (PJI) is a growing concern, especially with the increasing popularity of implant surgeries. PJI is one of the most severe complications in orthopedic surgery, with an incidence rate of around 5~10% [12]. For instance, the incidence rate of PJI after primary total knee arthroplasty is 1–2% [13]. Though antibiotics can somewhat reduce the infection rate, their efficacy on implant infections is not ideal, and systemic administration can lead to complications in vital organs such as the liver and kidneys. Moreover, due to the massive use of antibiotics, the problem of microbial resistance has become a common hazard that is difficult to resolve and has also become one of the most intractable challenges in the prevention and treatment of implant infection. Titanium alloy implants themselves have no bacteriostatic ability, and they quickly lead to the deposition, adhesion, and growth of bacteria after surgical implantation [14,15]. Although some researchers find that metal surface properties influence bacterial adhesion [16]. Therefore, new implant materials must have specific mechanical properties, biocompatibility, and bacteriostatic properties. For a long time, different surface modification approaches, such as surface charge and roughness, have been explored to improve the bacteriostasis of implants [17,18]. Recent research suggests that constructing specific nanostructured arrays on implant surfaces might be optimal for preventing bacteria [19,20]. The surface modification of titanium alloy improves its biomedical performance, such as by promoting bone growth and bacteriostatic properties [21]. Anodic oxidation is a traditional and effective surface treatment method that, after oxidation, lays a layer of titanium oxide film on the titanium alloy surface. The resulting film enhances the alloy’s wear and corrosion resistance [22,23] and has a certain level of biological activity [24]. In addition, the TiO_2_ nanotubes created via anodizing have a high specific surface area and strong adsorption capabilities, making them suitable as carriers for drug loading and release [25,26,27].

In this study, we designed three kinds of porous titanium alloys with different porosities and printed them by electron beam melting technology, and we obtained the ideal 3D printing structure through the mechanical properties testing of the samples. The TiO_2_ oxide layer was grown on the surface of a 3D-printed porous titanium alloy by anodic oxidation technology to improve the antibacterial property and the sample’s hydrophilicity.

## 2. Materials and Methods

### 2.1. Materials

In this study, we used several chemical reagents, including Acetone (A.R.) purchased from Rionlon Bohua (Tianjin, China) Pharmaceutical & Chemical Co., Ltd., Isopropanol (99%) purchased from Tianjin Heowns Biochemical Technology Co., Ltd. (Tianjin, China), Ethanol (A.R.) purchased from Tianjin Real&Lead Chemical Co., Ltd. (Tianjin, China), Hydrofluoric acid (40%) purchased from Tianjin Kermel Chemical Rea-gents Co., Ltd. (Tianjin, China), Nitric acid (A.R.) purchased from Tianjin Damao Chemical Reagent Factory (Tianjin, China), Ammonium fluoride (A.R.) purchased from Aladdin Reagents Co., Ltd. (Shanghai, China), and Ethylene glycol (98%) purchased from Tianjin Heowns Biochemical Technology Co., Ltd. (Tianjin, China). All the chemical reagents were used directly without further purification. Ti6Al4V was supplied by Arcam (Mölnlycke, Sweden), with a particle size of 45–105 μm.

### 2.2. Sample Preparation

Qingdao Weigao Medical Technology Co., Ltd. (Qingdao, China) provided the porous titanium alloy used in this experiment. They printed three models with different porosities and utilized computer-aided design by electron beam melting (Arcam EBM Q10Plus, Mölnlycke, Sweden). They use a computer-aided method to construct three models with different porosities. All three models were porous materials with the same surface and interior surface (10 mm in length and width, 1 mm in thickness). The difference between the three models lies in designing and printing three porous titanium alloys with different porosities by adjusting the wire diameter and element size, respectively denoted as 3D-60, 3D-75, and 3D-85. They used Ti6Al4V powder as a raw material to 3D-print the samples by electron beam melting. The 3D-printed porous titanium alloy for anodic oxidation was ultrasonically cleaned in acetone, isopropyl alcohol, ethanol, and deionized water successively for 15 min and then air dried [28]. Finally, the washed sample was pickled for 5 s within the mixed pickling solution (H.F., HNO_3_, deionized water volume ratio of 2:3:11) to remove the passivation layer on the surface and a small amount of unmelted metal powder in the 3D-printing process and improve the surface roughness [26]. (Note: the acid etching should be washed immediately with deionized water and anhydrous ethanol.) After ultrasonic cleaning with solvent and acid solutions, the samples were dried and used for anodic oxidation. 

Different nanostructures were prepared on the surface of a 3D-printed porous titanium alloy by the anodic oxidation technique. The electrolyte is a glycol solution containing 0.5 wt% ammonium fluoride and 1 v% deionized water. In a glycol solution serving as the electrolyte, The anode is the 3D-printed porous titanium alloy, and the cathode is the platinum plates. Both electrodes were kept at the same distance of 3 cm from each other in the same space. An adjustable constant-voltage D.C. power supply anodized the samples. Interestingly, a secondary anodizing technique was also employed wherein, after the first anodizing, the growing TiO_2_ oxide layer was removed using an ultrasonic process in deionized water and re-anodized under the same conditions.

### 2.3. Morphology and Contact Angle Characterization of the TiO_2_ Oxide Layer

We observed the morphological characteristics of the samples using a field emission scanning electron microscope (FE-SEM, S-4800, Tokyo, Japan). To determine the wettability of the samples, a contact angle measuring system (JC2000C1, Shanghai, China) employed the wettability at room temperature. For each measurement, distilled water drops were placed on a horizontal surface using a syringe, and the computer connected to a camera captured the resulting image. The contact angle was calculated by the numerical fitting of the droplet image, with each measurement repeated five times to ensure accuracy.

### 2.4. Bacteriostatic Test

For the antimicrobial activity testing, we chose the Gram-positive bacterium Staphylococcus aureus. Initially, a 5 mL L.B. liquid medium containing a 100 μL strain was shock-aerobically cultured overnight at 37 °C and then under the same conditions to dilute the bacterial solution for use. The samples were exposed to an ultraviolet lamp, placed in the bacterial solution, and continuously shaken at 180 rpm at 37 °C [20]. After being cultured for a specific time, take out the samples, and the O.D. values of each component of the bacterial solution were measured at 600 nm using an enzyme marker. The relative bacteriostatic rate was then computed using the following formula:

Antibacterial rate (%) = (A − B)/A × 100%

where A is the average O.D. value of the experimental group and B is the average O.D. value of the control group. To analyze the long-term antibacterial efficacy of the samples at different times, we chose 1, 3, 5, and 7 days as the time points to evaluate the OD values.

## 3. Results

### 3.1. Characteristics of 3D-Printed Porous Titanium Alloys

**Figure 1:**
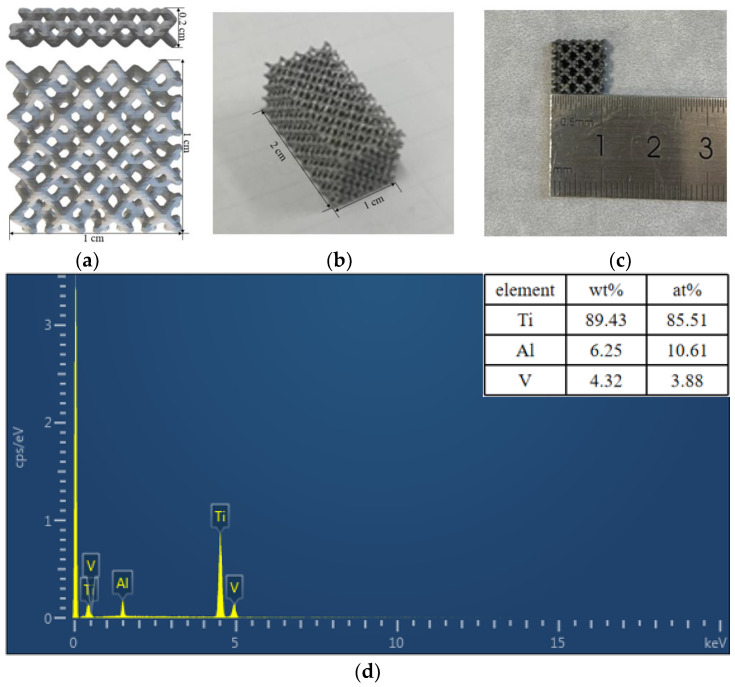
(**a**) Main and top views of the design sample; (**b**) Macrographs of 3D-printed porous Ti6Al4V specimens for the mechanical test, and (**c**) anodic oxidation; (**d**) Chemical compositions of the fabricated samples detected by EDS.

Figure 1a–c present a simulated diagram and a visible picture of porous scaffolds manufactured from Ti6Al4V powder. According to the simulated chart printed, there are two types of samples for oxidation and mechanical performance testing. The first type of sample had a length and width of 10 mm and a thickness of 1 mm, while the second type had a length and width of the same 10 mm and a thickness of 20 mm. The first sample type is for anodic oxidation, and the other model is for the mechanical properties tests. The three samples’ porosity was 60%, 75%, and 85%, respectively, denoted as 3D-60, 3D-75, and 3D-85.

Table 1 illustrates the variation in pore size across the three samples’ different porosity levels. Despite controversies in the literature concerning the most appropriate pore size and porosity, research suggests that the recommended pore size range is between 100 μm and 1000 μm. Different pore sizes serve distinct roles in bone ingrowth processes [29,30,31,32]. Prior studies have reported propensities ranging from 30% to 80% [33,34,35]. The three porosity and pore size configurations employed in this study are all within the appropriate range, as evidenced by the significant body of literature reviewed.

### 3.2. Mechanical Properties Evaluation

**Figure 2:**
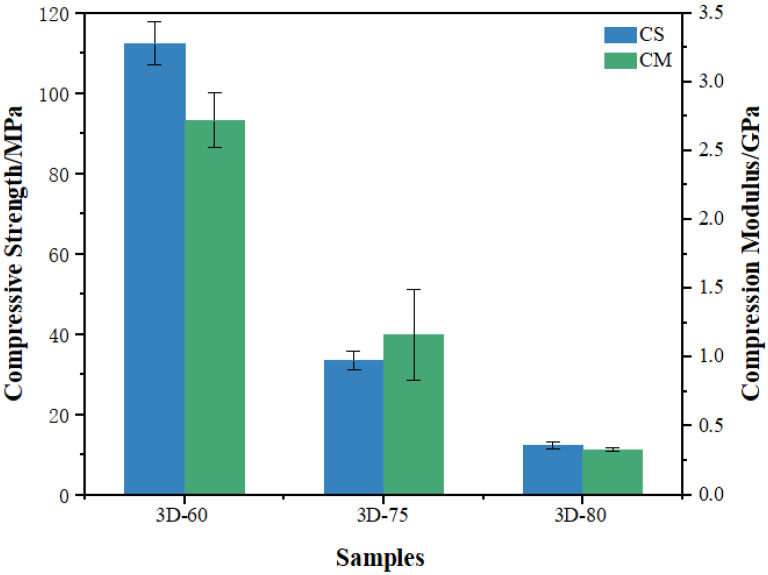
Mechanical properties of 3D-printed porous titanium alloys.

A compression test assesses the mechanical properties of porous Ti6Al4V scaffolds for medical implantation. Figure 2 illustrates the various samples’ compressive strength and compression modulus, which greatly varied and decreased as porosity increased. The sample 3D-60 with the biggest compressive strength is about 112 MPa and has the maximum elastic modulus. Further evaluation in Table 2 indicates that the sample models’ compressive modulus and yield strength declined concomitantly with increasing pore size. Calculate the elastic modulus for the three types of porous structures from 0.3 GPa to 2.7 GPa, which is much smaller than the traditional Ti6Al4V implant, which is about 110 GPa. We found that 3D-60 matches natural bone well, and the mechanical properties are much better than the other two samples. The greater the porosity and pore size, the more the samples’ mechanical properties are inferior. Therefore, we selected the 3D-60 model to further investigate modification, with a wire diameter of 5.0, a porosity of 60%, and a pore size of about 350 μm.

### 3.3. Morphological Characterization

**Figure 3:**
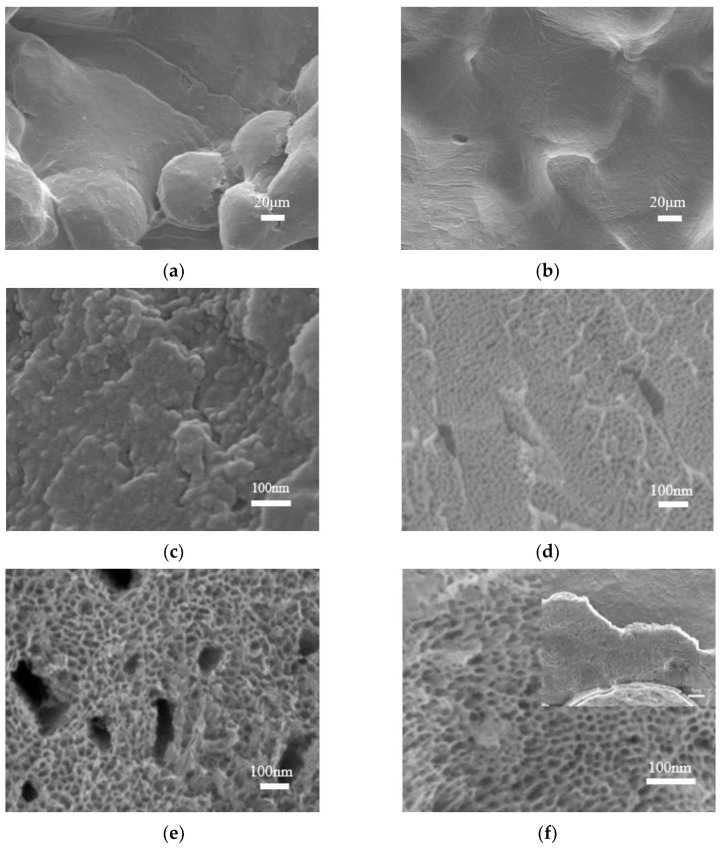
SEM images of (**a**) the original sample; (**b**) pre-treatment samples; and the titanium anodized at (**c**) 10 V, (**d**) 20 V, (**e**) 30 V, and (**f**) 40 V for 10 min.

Figure 3 presents high-magnification SEM images of the 3D porous titanium alloy and anodized specimens under bias potentials ranging from 10 V to 40 V after pre-treatment. Figure 3a,b displays the surface of the 3D porous titanium alloy after pre-treatment, where the passivated TiO_2_ oxide layer disappears and the surface becomes smoother, thus preventing the detachment of Ti6Al4V powder from the implant and its potential entrance into the human body. For low voltages, as shown in Figure 3c, only a thin TiO_2_ oxide layer forms on the surface of the titanium alloy. Increasing the anodizing voltage above 10 V produces a gradual change in the morphology of the anodized titanium alloy, from nanolayer (at 10 V) to nanopore (at 20 V) and then to nanotube (at 40 V), with a concomitant increase in pore size. However, high voltages may deteriorate the binding force between the TiO_2_ nanostructure and the titanium alloy, causing the oxide layer to fall off, as indicated in the inset of Figure 3f. Therefore, the optimal voltage for obtaining the desired nanoporous structure must balance the pore size and stability of the oxide layer.

**Figure 4:**
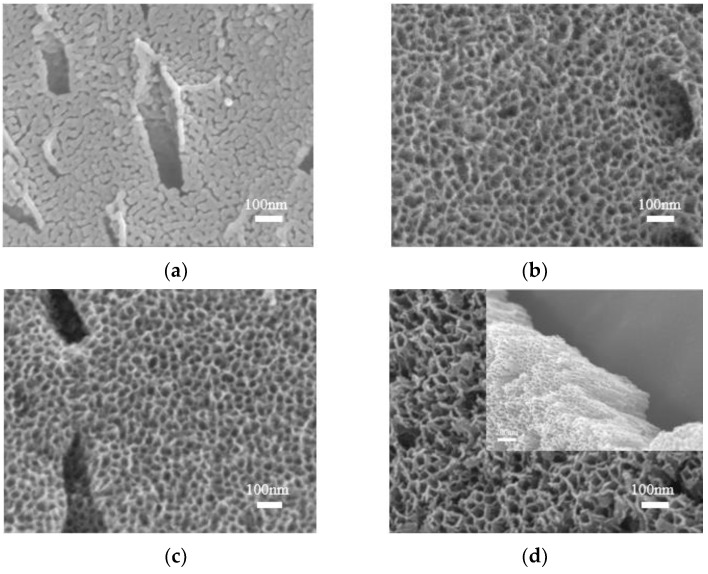
SEM images of 3D-printed porous titanium alloy anodized at 30 V with different times for (**a**) 5 min, (**b**) 10 min, (**c**) 20 min, and (**d**) 30 min.

To further investigate the effect of anodizing time on the TiO_2_ oxide layer, anodization was conducted at 30V for varying time durations. Figure 4 shows the morphology of the TiO_2_ oxide layer. It can be observed from the figure that the TiO_2_ oxide layer on the surface of the anodized titanium alloy gradually transformed into a nanotube with increasing anodizing time at the same voltage range. However, the bonding force between the TiO_2_ oxide layer and the matrix weakened after a certain point, leading to detachment.

Researchers have proposed several hypotheses and models for the formation mechanism of TiO_2_ nanotubes [35,36,37]. Our study, combined with these hypotheses, suggests that the formation mechanism of TiO_2_ nanotubes on the 3D-printed titanium alloy involves a process where an oxide layer initially forms on the surface of the alloy under the influence of voltage. Due to the inhomogeneity of the oxide layer, electrolytic F^−^ ions begin to etch small holes in the TiO_2_ oxide layer under the effect of the electric field, ultimately leading to the generation of tiny pores. Subsequently, the pores deepen and widen progressively, forming a porous layer with well-defined structures. High electric field intensity at the etched holes facilitates the process. Further reactions lead to the formation of a deep morphology pore, ultimately leading to the construction of the TiO_2_ nanotube structure [38,39]. 

**Figure 5:**
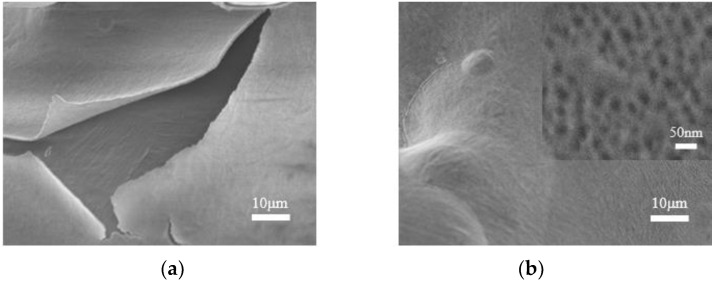
Low-power SEM images of primary (**a**) and secondary (**b**) anodizing under the same conditions.

Studies have shown that secondary anodization could improve the flatness of TiO_2_ nanotubes [26] and enhance the binding force between the TiO_2_ nanostructure and the matrix. In this study, after 30 V anodization for 20 min (3D-1), secondary anodization was conducted under identical conditions (3D-2), as shown in Figure 5. The results indicate that the bonding properties of TiO_2_ nanotubes to the substrate improve following the secondary anodization, in addition to increasing the flatness of the TiO_2_ nanotubes.

### 3.4. Wettability

**Figure 6:**
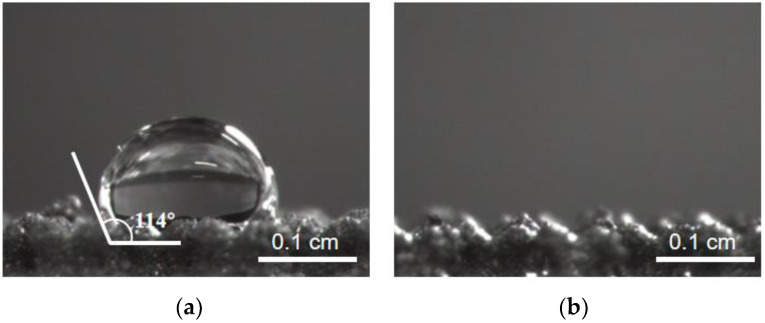
Digital pictures of water contact angles for (**a**) 3D-Ti and (**b**) 3D-Ti-2.

The surface wettability of implanted materials in vivo is a critical indicator of their moderate biological activity [36]. In general, materials with rougher surfaces exhibit better hydrophilicity [37]. As shown in Figure 6, the hydrophilic angle of the 3D-printed porous titanium alloy (3D-Ti) is significantly greater than 90°. However, applying a TiO_2_ nanolayer on the surface of the alloy after secondary anodization (3D-Ti-2) dramatically improved its hydrophilicity, as indicated by the close-to-zero hydrophilic angle. A more hydrophilic implant promotes the faster adsorption of various protein components, facilitating osseointegration and improving the success rate of implant operations [38].

### 3.5. Antibacterial Activity of 3D-Printed Porous Titanium Alloy with TiO_2_ Oxide Layer

The long-term bacteriostatic effects of the three samples of 3D-printed porous titanium alloy after primary anodizing (30 V, 20 min) and after secondary anodizing (twice, 30 V, 20 min) labeled as 3D-Ti, 3D-Ti-1, and 3D-Ti-2, respectively, were evaluated by conducting experiments. The results indicate that all three models exhibited a similar antibacterial trend towards Staphylococcus aureus for up to seven days. After a single day of culture, the antibacterial rate of 3D-Ti against *S. aureus* was approximately 8.5%. Moreover, after subjecting 3D-Ti to anodizing treatment, the antibacterial rate increased to 23% and 33.75%. This finding suggests that anodizing treatment has improved the antibacterial effect of 3D-printed porous titanium alloy. A 7-day long-term bacteriostatic evaluation indicates a significant improvement in long-term bacteriostatic activity after two anodic oxidation surface treatments.

**Figure 7:**
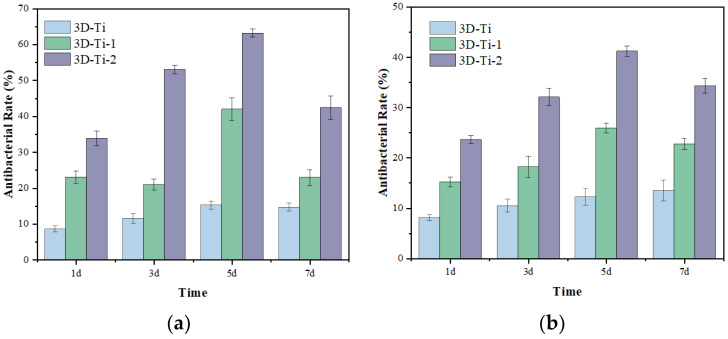
The antibacterial activity of samples at different times in (**a**) *S. aureus* and (**b**) *E. coli*.

Although infections caused by bone implant material 3 months after surgery are usually caused by *S. aureus* [40], we also tested for bacteriostasis with *E. coli*. As shown in Figure 7b, it can be found that the antibacterial trend of the samples against the two bacteria is consistent, and the long-term antibacterial activity of the samples has been improved to a certain extent after secondary anodizing. At the same time, it was found that Staphylococcus aureus was more likely to be killed, possibly due to differences in cell walls [17], but the specific reasons for this should be the subject of our follow-up research.

## 4. Conclusions

In this study, aimed at the problems of high elastic modulus and easy infection after implantation of titanium alloy bone implants, we designed and prepared porous titanium alloy antibacterial structures with different porosities using 3D printing. At the same time, the anodizing modifies the surface of the 3D-printed porous titanium alloy to integrate the structural design and surface modification organically. Three kinds of porous titanium alloys with different porosities (60%, 75%, and 85%) were prepared with 3D printing technology. Compression experiments measured their mechanical properties and modified them by anodic oxidation of the porous titanium alloys (porosity 60% of the sample) with excellent structure. The growth mechanism of TiO_2_ nanolayers on a porous titanium alloy surface was investigated by adjusting the voltage and time of anodizing. A TiO_2_ nanotube of uniform size was grown on the sample’s surface by secondary anodizing to improve the hydrophilicity and antibacterial properties of the model. Although the bacteriostatic performance of the sample obtained in this paper needs to be further enhanced, it lays the foundation for future clinical applications.

## Figures and Tables

**Table 1:** Samples of aperture measurement data.

Samples	3D-60	3D-75	3D-85
Pore sizes (μm)	353 ± 23	563 ± 38	915 ± 41

**Table 2:** Mechanical property parameters of 3D-printed porous titanium alloy.

Samples	Max Load KN	Yield Strength MPa	Compressive Strength MPa	Compression Modulus GPa
3D-60	11.86 ± 0.54	97.79 ± 5.43	112.48 ± 5.23	2.72 ± 0.2
3D-75	3.6 ± 0.27	30.4 ± 1.66	33.56 ± 2.25	1.16 ± 0.04
3D-85	1.35 ± 0.1	10.28 ± 0.74	12.37 ± 0.94	0.33 ± 0.01

## Data Availability

Data is contained within the article.
